# The Assembly-Disassembly-Organization-Reassembly Mechanism for 3D-2D-3D Transformation of Germanosilicate IWW Zeolite**

**DOI:** 10.1002/anie.201400600

**Published:** 2014-05-13

**Authors:** Pavla Chlubná-Eliášová, Yuyang Tian, Ana B Pinar, Martin Kubů, Jiří Čejka, Russell E Morris

**Affiliations:** Department of Synthesis and Catalysis, J. Heyrovský Institute of Physical Chemistry, Academy of Sciences of Czech Republicv.v.i. Dolejškova 3, 18223 Prague (Czech Republic)http://www.jh-inst.cas.cz; EaStCHEM School of Chemistry, University of StAndrews, St. Andrews KY16 9ST (UK); Laboratory of Crystallography, ETH ZürichWolfgang-Pauli-Strasse 10, CH-8093 Zürich (Switzerland)

**Keywords:** ADOR, germanosilicate, IWW, solid-state transformation, zeolites

## Abstract

Hydrolysis of germanosilicate zeolites with the IWW structure shows two different outcomes depending on the composition of the starting materials. Ge-rich IWW (Si/Ge=3.1) is disassembled into a layered material (IPC-5P), which can be reassembled into an almost pure silica IWW on treatment with diethoxydimethylsilane. Ge-poor IWW (Si/Ge=6.4) is not completely disassembled on hydrolysis, but retains some 3D connectivity. This structure can be reassembled into IWW by incorporation of Al to fill the defects left when the Ge is removed.

In recent papers we reported a synthetic route to lamellar or 2D zeolites using the chemically selective disassembly of a 3D parent zeolite with the **UTL** structure^1^, ^2^ and subsequently showed how new, fully tetrahedral zeolitic materials could be prepared by reassembling the 2D layers into 3D solids with new topologies. We describe this process using the ADOR (assembly-disassembly-organization-reassembly) acronym.^3^ In this way, two zeolites were prepared: IPC-2^3^ (which is isostructural to COK-14^4^ with the IZA code^5^ **OKO**) and IPC-4^3^ (code **PCR**).

We proposed that for successful application of the ADOR mechanism to other parent zeolite structures, the following structural features are required: 1) the presence of double-four-ring units (*D4R*) and 2) that these *D4R* units are preferentially occupied by Ge.^6^–^8^ Herein, we describe the application of the ADOR mechanism for the 3D-2D-3D transformation of another zeolite with the **IWW** framework type^9^ comprising layers separated by *D4R*s.

Three batches of IWW zeolite were prepared. All hydrolysis reactions were completed on two samples—one with high content of germanium (Si/Ge 3.1), designated as Ge-rich IWW, and one with low content of germanium (Si/Ge 6.4), Ge-poor IWW. A further batch of sample with Si/Ge=3.6 was prepared for structural studies using synchrotron X-ray diffraction.

A detailed study of germanium location in zeolite ITQ-22 (**IWW**) is necessary to understand the structural changes occurring during the ADOR process, especially for samples differing in the content of Ge. IWW with Si/Ge 3.6 and 6.4 were investigated by diffraction techniques. Rietveld refinements of high-resolution synchrotron powder diffraction data were performed in the space group *Pba*2. The location of the germanium atoms in the structural models was determined by careful analysis of the average interatomic distances and Fourier analysis, and showed that the only sites with significant Ge occupancy were in the *D4R* units. The average D4R unit in the Ge-rich sample has 6 Ge and 2 Si atoms [6Ge,2Si]. In contrast, the Ge-poor IWW has an average site occupancy of near [4Ge,4Si] for each *D4R.* For detailed experimental methods and results and discussion see the Supporting Information.

The effect of hydrolysis under acidic conditions was investigated. Figure 1 shows the XRD pattern of Ge-rich **IWW** hydrolyzed in 0.1 m HCl. The most noticeable change is a disappearance of the 111, 211, and 311 peaks, consistent with order in the *c* direction being reduced. The positions of peaks without contribution in the *c* direction are unchanged, for example, 200, 400, and 310. A new, broader peak 001, which can be attributed to the stacking of **IWW** layers along the *c* axis, is seen between 2*θ*=7.65 and 8.78° depending on the exact hydrolysis conditions. The hydrolysis process leads to a reduction of the interlayer distance by between 1 and 3 Å. The removal of most Ge atoms was confirmed by both chemical analysis (Si/Ge increased from 3.1 up to 45.9) and by solid state ^29^Si MAS NMR where the signal for Si Q^4^ connected to at least 1 Ge atom was no longer detected. We denote the hydrolyzed Ge-rich **IWW** as IPC-5P, a new lamellar material with IWW structure of the layers (Scheme 2).

**Figure 1:**
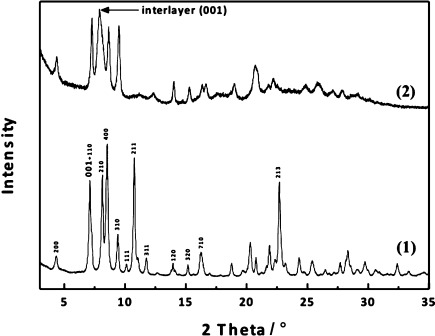
XRD powder patterns of IWW and Ge-rich IWW (1) hydrolyzed in 0.1 m HCl at room temperature for 43 h (2).

**Scheme 1:**
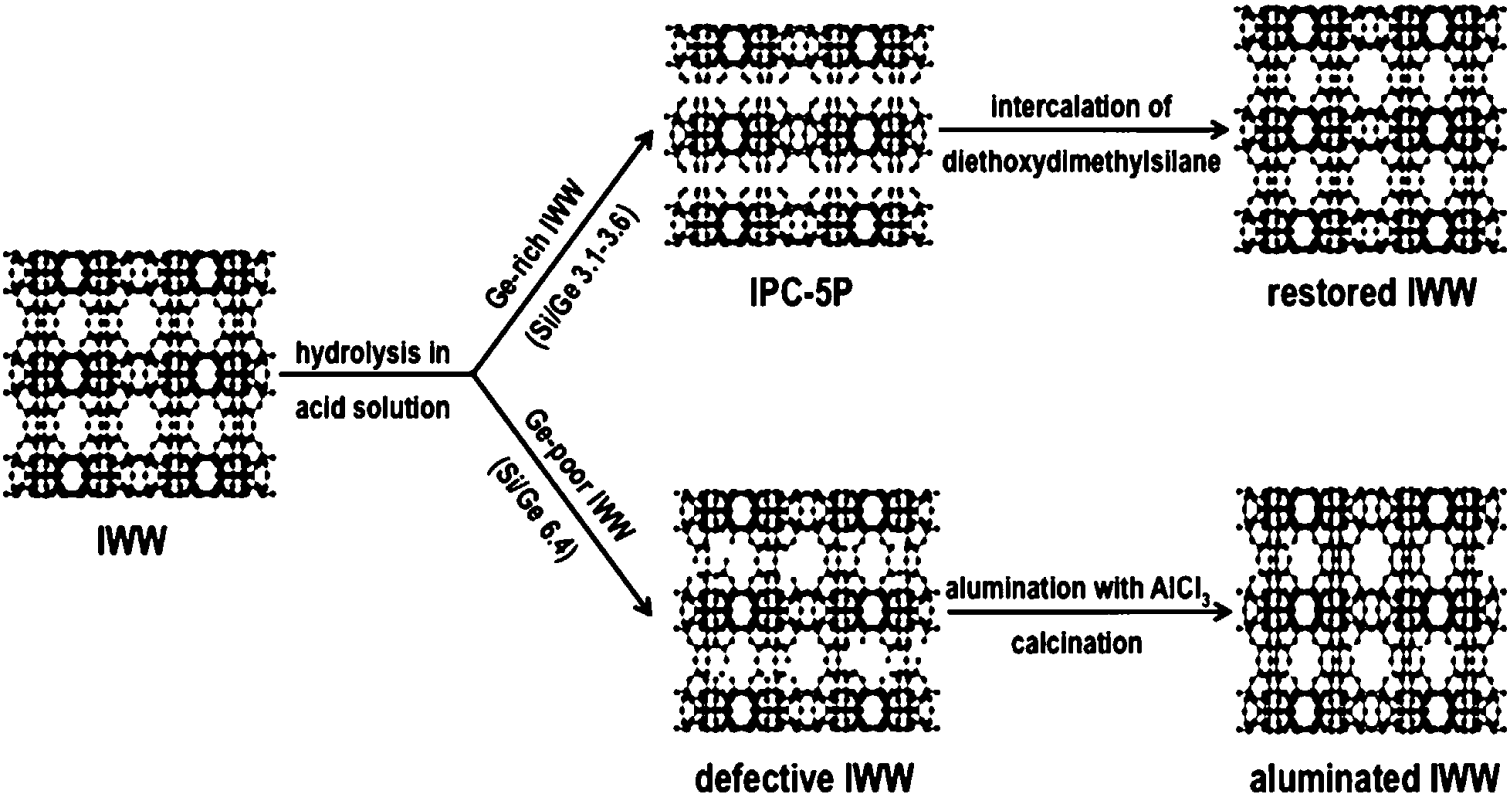
Hydrolysis of IWW with different germanium content and post-synthesis treatments leading to restored IWW frameworks with different chemical composition. Aluminum atoms are shown as green dots.

Hydrolysis of Ge-poor **IWW**, on the other hand, did not lead to such distinctive structural changes. Figure 2 compares the XRD patterns of Ge-rich **IWW** and Ge-poor **IWW** hydrolyzed under the same conditions. Hydrolyzed Ge-poor **IWW** does present a diffraction pattern that is distinct from the parent IWW, showing that some chemical changes have occurred. However, there are no new peaks that can be assigned to a particular ordering of the IWW layers, and—despite the changes in intensity—peaks such as the 111 and 211 remain visible. This suggests that despite the extraction of germanium from *D4R*s the material has not been fully hydrolyzed into layers and that connections are still present that hold the structure together forming a defective IWW-like material (Scheme 2). The Si/Ge ratio increased from 6.4 to 50.9 and 121.5 for hydrolysis in 0.1 m and 12 m HCl, respectively.

**Figure 2:**
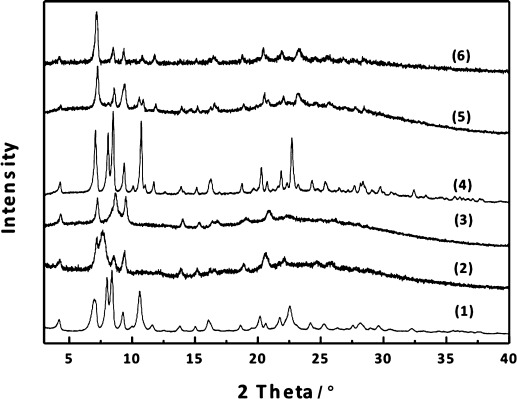
XRD pattern of calcined Ge-rich IWW (Si/Ge 3.1) (1), its hydrolyzed forms after treatment with 0.1 m HCl (2) or 12 m HCl (3), and Ge-poor IWW (Si/Ge 6.4) (4) and its hydrolyzed forms after treatment with 0.1 m HCl (5) or 12 m HCl (6).

To verify full separation of the layers in IPC-5P and the possibility of further manipulation, we attempted a swelling treatment using the hexadecyltrimethylammonium cation.^10^ Generally, when swelling is successful and the surfactant is stacked roughly perpendicular to the layers, we expect a shift of the 001 peak to significantly lower 2*θ* values.

In the surfactant-treated sample, denoted IPC-5SW, the interlayer reflection 001 (in IPC-5P at 2*θ*=7.8°) is shifted to lower 2*θ* values and now overlaps with the peak at 2*θ*=7.2° (see the Supporting Information). Overall, due to the relatively small shift of the 001 reflection, we believe that the surfactant is not stacked perpendicular to the layers but horizontally between them. New reflections in the 2*θ* range 10–35° also appeared, which are marked with asterisks in Figure 3 (3). The positions of these peaks closely correspond to those in the parent **IWW** structure, as the rearrangement of the material reformed connections between the layers that were lost on hydrolysis. Since the relatively high pH used is known to promote Si—O bond making and breaking, this is not an entirely unexpected result. As a side effect a part of IWW layers may be dissolved and transformed into mesoporous particles of M41S type.^11^ This can be seen in the low-angle X-ray diffraction region as a broad reflection (See Figure S7.)

**Figure 3:**
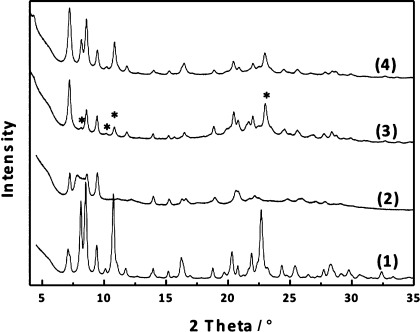
XRD patterns of Ge-rich IWW (1), hydrolyzed IPC-5P (2), surfactant-treated IPC-5SW (3), and calcined surfactant-treated-intercalated material—restored IWW (4). The asterisks mark some of the reflections newly appeared after swelling treatment.

However, when a part of the “surfactant-treated” IPC-5SW was calcined, the structure collapsed and we observe only negligible intensities in the 2*θ* range 6–35°, suggesting that the rearrangement has not formed enough connections to lead to a stable material. The broadened peak in the low angle area may indicate the formation of mesoporous particles. To include more silicon and increase the number of interlayer connections IPC-5SW was treated with diethoxydimethylsilane (DEDMS). After calcination the material (which we denote as “restored” IWW) shows a very similar XRD pattern to the parent IWW. Removing most Ge atoms from the framework and replacing them by Si atoms decreased the unit cell size slightly. A chemical analysis confirmed the removal of Ge when Si/Ge ratio increased from 3.1 up to 73.4.

An attractive possibility is to restore **IWW** not with new Si from the DEDMS but with other dopant atoms, such as aluminum. Attempts to incorporate Al into defects were carried out on both hydrolyzed Ge-rich and Ge-poor samples (Figure 4) using AlCl_3_ as the source of Al. The aluminated Ge-rich material displays peaks of low intensity (Si/Ge ratio increased up to 102). In contrast, the aluminated Ge-poor **IWW** shows the same architecture as the parent **IWW**. We explain the differences in alumination of Ge-rich and Ge-poor hydrolyzed **IWW** as follows: When the *D4R* units are destroyed during the hydrolysis, as is the case of Ge-rich sample, we cannot rebuild the interlayer connections using only aluminum atoms as this would break Lowenstein’s rule.^15^ However, in the Ge-poor **IWW** the hydrolysis led only to defects in the *D4R*s. In that case aluminum can be incorporated into the defects and thus restore a **IWW** structure with new chemical composition (Si/Ge 115 and Si/Al 27; Scheme 2).

**Figure 4:**
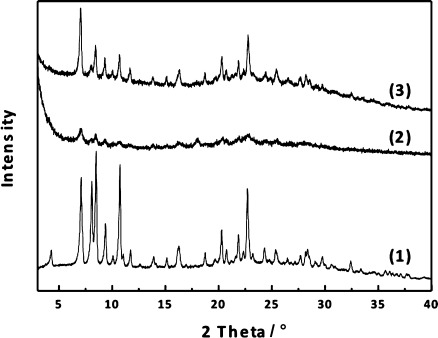
XRD patterns of calcined IWW (1) in comparison with aluminated hydrolyzed Ge-rich IWW (2) and aluminated hydrolyzed Ge-poor IWW (3).

The structural changes were also evaluated based on argon (Figure 5 A) and nitrogen (see the Supporting Information) sorption isotherms. Parent **IWW** (Figure 5 A (1)) is a typical microporous zeolite with BET surface area of 416 m^2^ g^−1^ and micropore volume of 0.169 cm^3^ g^−1^. The pore size distribution shown in Figure 5 B,C is centered at 0.65 nm corresponding to the presence of a 12-10-8-R channel system.

**Figure 5:**
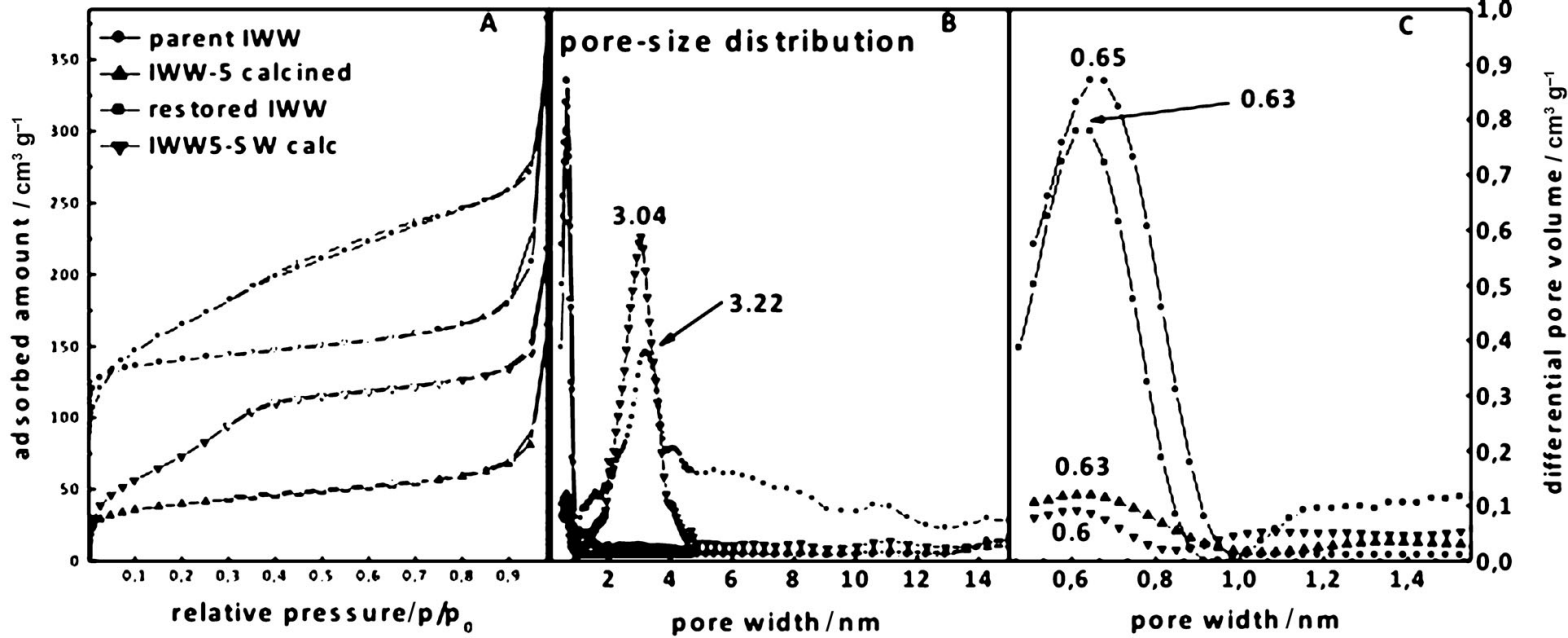
A) Argon adsorption isotherms measured at −186 °C. Parent calcined IWW zeolite (Si/Ge 3.1) (1), hydrolyzed and calcined IPC-5 (2), surfactant-treated and calcined IPC-5SW (3), and surfactant-treated-intercalated and calcined—restored IWW (4). Open symbols denote desorption isotherms. B,C) Pore size distribution in the range between 0-15 nm (B), a closer view on the range 0.5–1.5 nm (C). The samples were hydrolyzed using 0.1 m HCl.

After hydrolysis (to form IPC-5 after calcination) the surface area as well as micropore and total pore volume are greatly reduced (Table 1). Such behavior corresponds to a reduction in micropore volume through the destruction and removal of the *D4R*s, which destroys pores parallel to the layers. However, the layers still possess 12-8-R channels going through the layers and the material still retains some micropore volume (0.030 cm^3^).

**Table 1:** Textural properties of IWW and its post-synthesis modified forms evaluated from argon adsorption measured at −186 °C.

	Si/Ge			Argon adsorption		
	(EDX)		BET [m^2^ g^−1^]	*V*_mic_^[a]^ [cm^3^ g^−1^]	*V*_tot_^[b]^ [cm^3^ g^−1^]
Parent IWW	3.1		416.1	0.169	0.484
Hydrolyzed (IPC-5)	45.86		123.6	0.030	0.213
Surfactant-treated (IPC-5SW calcined)	–		278.5	0.015	0.278
Restored IWW	73.38		509.5	0.146	0.450

[a] Micropore volume. [b] Total pore volume.

“Swelling” of the IPC-5P structure, followed by calcination, leads to increased total surface area but this is primarily due to the formation of mesoporous particles. Hence, in the IPC-5SW isotherm (Figure 5 A (3)) the continuous uptake of argon in the *p*/*p*_0_ range 0.02–0.40 is due to the filling of small mesopores, which are also evidenced by pore size distribution analysis (a large band centered around 3 nm). After intercalation of DEDMS and restoration of the **IWW** structure the filling of micropores takes place in the low relative pressure region and then continues in the filling of small mesopores. The BET area increased up to 510 m^2^ g^−1^ as the whole 12-10-8-R channel system was rebuilt. This is higher than the parent IWW but is due to the presence of additional mesoporous particles not present in the parent material. The micropore volume, which is a better measure of the microporosity in the samples, is 0.146 cm^3^ g^−1^ in the restored **IWW**, close to the original value for parent **IWW** (0.169 cm^3^ g^−1^). The pore size distribution analysis revealed an intensive band centered at 0.63 nm near its original position for the parent **IWW** (0.65 nm) as well as broader maxima coming from adsorption into the mesoporous particles.

To confirm the structural changes in **IWW** during hydrolysis and restoration of the **IWW** structure ^29^Si MAS NMR spectra were collected (Figure 6 A). The parent **IWW** (Si/Ge 3.1) exhibits two separated resonances, at −110 pm and −113 pm. The signal at −113 pm is assigned to pure Si Q^4^ groups while the signal at −110 ppm corresponds to Si Q^4^ connected to at least 1 Ge atom.^12^ After hydrolysis the resonance at −110 ppm disappeared.

**Figure 6:**
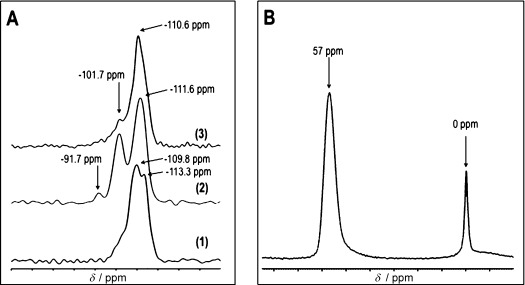
A) ^29^Si MAS NMR data for parent Ge-rich IWW (1), layered IPC-5P (2), and final restored IWW structure (3). B) ^27^Al MAS NMR data for aluminated sample prepared from hydrolyzed Ge-poor IWW.

In addition, a new resonance is observed around −102 ppm, which is commonly assigned to Q^3^ signals. There is also a small Q^2^ signal around −92 ppm. After restoration of the **IWW** structure the Q^3^ signal is reduced in intensity consistent with the restoration of a fully condensed **IWW**.

The ^27^Al MAS NMR spectrum was collected for **IWW** restored by alumination of hydrolyzed Ge-poor **IWW** (Figure 6 B). A major resonance at 57 ppm indicates the existence of tetrahedrally coordinated Al in the framework while the minor signal at 0 ppm comes from a small amount of octahedrally coordinated Al.^13^ The presence of a resonance for tetrahedrally coordinated Al is clear evidence that some Al has been incorporated into the framework of the solid, although the presence of extra-framework (octahedrally coordinated) Al shows that not all of the Al is incorporated into the framework.

In conclusion, the location and amount of Ge in the *D4R*s of **IWW** significantly influence the structural stability in acidic environment. The hydrolysis results for Ge-rich and Ge-poor **IWW** materials show two possible outcomes. Full hydrolysis of Ge-rich materials resulted in a 2D solid (IPC-5P). The layered IPC-5P was converted back to the 3D structure of IWW by incorporation of silylating agent. The restored **IWW** has a significantly higher Si/Ge ratio (73) than the parent **IWW** (3.1).

The hydrolysis of Ge-poor **IWW** also led to extraction of germanium from mixed Si/Ge *D4R* units. However, because there is much less Ge in the *D4R* than for the Ge-rich sample, the material preserved enough interlayer connections to retain its 3D **IWW** framework, albeit with significant defects. Moreover, the structural defects can be filled by incorporation of Al into defined crystallographic positions.^14^

Our work confirms the application of the ADOR mechanism on another zeolitic structure—**IWW**. In contrast to zeolite UTL where we obtained two new zeolites (OKO, PCR), here the layered structure IPC-5P strongly tends to reform the original **IWW** framework.

## Experimental Section

The preparation of **IWW** followed the procedure described in Ref. 9. The calcined **IWW** samples were treated in different acids (HCl, HNO_3_, CH_3_COOH) of various concentrations (0.1–12 m) between ambient temperature and up to 100 °C. The hydrolyzed material was treated with C_16_TMA surfactant and subsequently intercalated with DEDMS and calcined. The hydrolyzed solid was also directly intercalated with DEDMS or octylamine and calcined. The solid materials were characterized by X-ray powder diffraction, nitrogen and argon sorption, solid state NMR spectroscopy (^29^Si MAS NMR, ^27^Al MAS NMR), and EDX. Details of synthesis, subsequent modifications, and spectroscopic characterization are described in the Supporting Information.
