# Supplementary material for: The Assembly-Disassembly-Organization-Reassembly Mechanism for 3D-2D-3D Transformation of Germanosilicate IWW Zeolite
Source: Angew Chem Int Ed Engl. 2014 May 13;53(27):7048–52. doi: 10.1002/anie.201400600 (PMC4320762; doi:10.1002/anie.201400600)
Supplement: Supplementary file 1 [file anie0053-7048-sd1.pdf]

Angewandte Chemie
Eine Zeitschrift der Gesellschaft Deutscher Chemiker

Supporting Information
© Wiley-VCH 2014
69451 Weinheim, Germany

The Assembly-Disassembly-Organization-Reassembly Mechanism for 3D-2D-3D Transformation of Germanosilicate IWW Zeolite**

Pavla Chlubná-Eliášová, Yuyang Tian, Ana B. Pinar, Martin Kubů, Jiří Čejka, and Russell E. Morris**

anie_201400600_sm_miscellaneous_information.pdf

Supporting Information

Experimental section 3

Characterization 4

Structure Analysis 5

List of Tables and Figures:

Table S1 Examples of conditions used for hydrolysis of Ge-rich IWW (Si/Ge ratio 3.1)

Table S2 Textural properties of IWW samples valuated from argon and nitrogen sorption

Table S3 Powder diffraction data collection parameters for Ge-rich and Ge-poor **IWW** samples

Table S4 Crystallographic data from the Rietveld refinement of Ge-rich **IWWa**

Table S5 Atomic parameters for Ge-rich **IWWa**[,b]

Table S6 Selected interatomic distances (Å) for Ge-rich **IWWa**

Table S7 Selected bond angles (º) for Ge-rich **IWW**

Table S8 Crystallographic data from the Rietveld refinement of Ge-poor **IWWa**

Table S9 Atomic parameters for Ge-poor **IWW** (55)[a,b]

Table S10 Selected interatomic distances (Å) for Ge-poor **IWW** [a]

Table S11 Selected bond angles (º) for Ge-poor **IWW**

Fig. S1 The framework structure of ITQ-22 (**IWW** framework type). (a) The [010] projection showing the double-four-rings (T atoms represented with balls); (b) the [001] projection showing the 12- and 8-ring channels and (c) T atoms in the double-four-rings. Oxygen atoms have been omitted for clarity.

Fig. S2 Fourier differences map for Ge-rich **IWW** with all T atoms as Si. Electron density clouds appear on top of the eight T sites in the *d4R*, indicating the presence of Ge.

Fig. S3 The observed (black), calculated (red) and difference (blue) profiles for the Rietveld refinement of Ge-rich **IWW**. The data in the inset have been scaled up by a factor of 6 to show more detail at high angles. The tick mark indicate the positions of the reflections.

Fig. S4 Background correction for the powder diffraction pattern of the Ge-poor **IWW**. It is an interpolation of points selected by eye and adjusted manually during the course of the refinement. The humps under the two peaks at *ca*. 6 º correspond to an unidentified impurity that was included in the background so it does not disturb the refinement.

Fig. S5 The observed (black), calculated (red) and difference (blue) profiles for the Rietveld refinement of the Ge-poor **IWW**. The data in the inset have been scaled up by a factor of 6 to show more detail at high angles. The tick marks indicate the positions of the reflections.

Fig. S6 XRD patterns of IPC-5P samples prepared by hydrolysis of Ge-rich IWW (Si/Ge 3.1) under various conditions described in **Table S1**

Fig. S7 Small-angle (**A**) and wide-angle (**B**) XRD patterns of parent Ge-rich **IWW** (Si/Ge 3.1) **(1)**, hydrolysed IPC-5P **(2)**, its surfactant-treated IPC-5SW **(4)**, and the surfactant-treated material intercalated with diethoxydimethylsilane and calcined, restored IWW **(6)**. Part of the hydrolysed sample was calcined (IPC-5) **(3)**, as well as part of the surfactant-treated sample (IPC-1SW calc) **(5)**.

Fig. S8 XRD patterns of hydrolysed Ge-rich **IWW (1)**, after intercalation treatment with octylamine (calcined) **(2)**, and after direct intercalation of diethoxydimethylsilane (**calcined) (3**).

Fig. S9 Adsorption isotherms of nitrogen measured at 77K (left) and argon measured at 87K (right). Parent calcined **IWW** zeolite (Si/Ge 3.1) **(1)**, hydrolysed and calcined IPC-5 **(2)**, surfactant-treated and calcined IPC-5SW calc **(3)**, and surfactant-treated-intercalated and calcined, restored **IWW (4)**. The open points denote desorption. Although the N_2 and Ar isotherms show the same trend for each sample, the values of their BET surface area, micropore volume and others can slightly differ, which is caused by different parameters of both adsorptives (see results in **Table S2**).

Experimental section

The preparation of IWW followed the procedure in the reference[1]. The organic structure directing agent (OSDA), 1,5-bis-(methylpyrrolidinium)pentane (MPP) cation, was synthesized by reacting 20g of N-methylpyrrolidine (97%, Aldrich) and 18.7g of 1,5-dibromopentane (97%, Aldrich) in 300ml of acetone (99%, Fisher Chemical) under reflux for 24 hours. The hydroxide form of $MPP(OH)_2$ was obtained by anion-exchange with Ambersep 900 (Acros) or AG 1-X8 resin (Bio-Rad) and concentrated to an aqueous solution (13.6 wt%). Typically, for Ge-rich IWW (with the final Si/Ge molar ratio 3.1-3.6), 2.750g of tetraethyl orthosilicate (TEOS, 98%, Aldrich) and 0.691g of GeO_2 (99.9999%, Alfa Aesar) were added to 10g of $MPP(OH)_2$ solution under continuous stirring. The resulting mixture was vigorously stirred to evaporate the ethanol formed by the hydrolysis of TEOS and the excess water, forming a final gel composition of $0.66SiO_2$: $0.33GeO_2$: $0.25MPP(OH)_2$: $3.5H_2O$. The composition of the reaction gel for Ge-poor **IWW** (with final Si/Ge molar ratio 6.4) was as followed: $0.66SiO_2$: $0.1GeO_2$: $0.25MPP(OH)_2$: $3.5H_2O$. The gel was heated in Teflon-lined stainless steel autoclaves at 175 °C for 11 days. The final solid was isolated by filtration, washed with water and dried at 100 °C overnight. The resulting solid was calcined at 580 °C for 6 hours in air.

Hydrolysis was carried out with calcined samples in different acids (HCl, HNO_3, CH_3COOH) of various concentrations (0.1-12M) at the temperature from ambient up to 100 ˚C. The example of hydrolysis conditions are shown in **Table S1**. Typically, 0.1g of solid was stirred with 100-250 ml of acid solution for 16-48 hours. The solid was centrifuged and dried at 60-100 °C overnight. A part of the hydrolysed material was calcined at 550 °C for 6 hours.

Intercalation of hydrolysed IWW

Swelling: Swelling was performed using a mixture of C_{16}TMA-Cl (25%) and C_{16}TMA-OH (25%) with the ratio for 1 g of hydrolysed **IWW** (IPC-5P) 6.7ml of C_{16}TMA-Cl (25%) and 13.3ml of C_{16}TMA-OH (25%). C_{16}TMA-OH (25%) was prepared from 25% solution of C_{16}TMA-Cl by ion-exchange using resin AG 1-X8 (Bio-Rad). The slurry was stirred in the ice-cooled bath for 7 hours. The product was isolated by centrifugation, properly washed with water and dried at 60° C. A part of the swollen sample was further calcined at 650 °C for 6 hours.

Intercalation of diethoxydimethylsilane (DEDMS): The hydrolysed IPC-5P or the surfactant-treated IPC-5P was treated with DEDMS in 1M HNO_3 solution (for 1 g zeolite, 0.5 g of DEDMS and 10 g of acid). The reaction was carried out in the autoclave at 175 °C for 16 hours. The solid was filtered, washed with water and calcined at 650 °C for 6 hours.

Reaction with octylamine: Hydrolysed IPC-5P was stirred with octylamine (for 1g used 6.5g of octylamine) at 75 °C for 16 hours. The solid was separated by centrifugation, dried at 40 °C and finaly calcined at 650 °C for 6 hours.

Alumination: Alumination was performed by reacting 0.2g of $AlCl_3$ with 0.1g of hydrolysed IWW in 50 ml of 0.05M HCl solution under reflux for 3 days. The solid was isolated by centrifugation, washed with water, dried overnight and calcined at 550 °C for 6 hours.

Characterisation

The structure and crystallinity of zeolites under study were determined by X-ray powder diffraction using a Bruker AXS D8 Advance diffractometer equipped with a graphite monochromator and a position sensitive detector Våntec-1 using CuKα radiation in Bragg–Brentano geometry or STOE diffractometer under CuKα radiation in Debye-Scherrer mode. For the structure analysis of **IWW** powder diffraction data were collected on the Materials Science-Powder X04SA Beamline at the Swiss Light Source (SLS) at Paul Scherrer Institut in Villigen, Switzerland[2]. Data collection details are given in **Table S3**.The Rietveld refinement was performed using the program package XRS−82[3], and the structure drawings were produced using the program CrystalMaker[4]. The plots with observed, calculated and differences patterns were prepared with the programme ppp14[5].

The chemical analysis was obtained by the Energy Dispersive X-Ray Spectroscopy (EDX) on a Jeol JSM 5600 instrument.

Solid-state NMR spectra were acquired using Bruker Avance III 600 MHz spectrometer equipped with a widebore 14.1 T magnet. Powdered samples were packed into conventional 4 mm ZrO2 rotors. ^{29}Si MAS NMR spectra were obtained using single-pulse experiments, with a recycle interval of 180 s. The MAS rate was 10 kHz. For the ^{27}Al MAS NMR the MAS rate of 14 kHz was used. The IR spectra were collected by a Perkin Elmer Spectrum GX IR spectrometer from the wave number range of 400-4000 cm-1.

Adsorption isotherms of nitrogen (at -196˚C) and argon (at -186˚C) were measured on a Micromeritics ASAP 2020 static volumetric instrument. In order to attain sufficient accuracy in the accumulation of the adsorption data, the ASAP 2020 was equipped with pressure transducers covering the 133 Pa, 1.33 kPa and 133 kPa ranges. Prior to the sorption experiments, samples were outgassed at 110 °C under turbomolecular pump vacuum until the residual pressure of 0.5 Pa was obtained. After further heating at 110 °C for 1 h the temperature was increased (1 °C/min) until the temperature 300 °C was achieved. This temperature was maintained for 6 h. The specific surface area (SBET) was evaluated by BET method[6] using adsorption data in the p/p_0 range of 0.05-0.20. The adsorbed amount at relative pressure p/p_0 = 0.98 reflects the total adsorption capacity (V_{tot}). For adsorption isotherms of nitrogen, the t-plot method[7] was applied to determine the volume of micropores (V_{mic}). For adsorption isotherms of argon, the DFT algorithm (using standard Micromeritics software for cylindrical pores for Argon on Oxides at -186 °C) was used to calculate the volume of micropores (Vmic) and pore-size distribution for pores less than 5 nm in diameter. The volume and pore-size distribution of mesopores with the size from 5 to 20 nm was calculated from the desorption branch of the isotherm using BJH method[8] with Halsey equation.

Structure analysis

The diffraction pattern of Ge-rich **IWW** (Si/Ge molar ratio 3.6) and Ge-poor **IWW** (Si/Ge molar ratio 6.4) correspond to zeolite ITQ-22 (structure type **IWW**). They were indexed with the programme TREOR[9] implemented in the software CMPR[10] in an orthorhombic unit cell with parameters a = 42.01, b = 12.95, c = 12.64 Å, for Ge-rich **IWW**, and a = 42.01, b = 12.96, c = 12.61 Å, for Ge-poor **IWW**. The systematic absences were indicative of the extinction symbol *Pba−* (space groups *Pbam* and *Pba2*). Structure refinement of the diffraction data of Ge-rich **IWW** was started in *Pbam*, in which the structure was initially solved1, using those coordinates as the initial model structure. Geometric restraints were imposed on the bond distances and angles of the framework atoms throughout the refinement, and their weight relative to the diffraction pattern was decreased as the refinement progressed. Neutral scattering factors were employed for all atoms. All the tetrahedrally coordinated atoms (T atoms) were initially refined as pure Si. At the first stages of the refinement, the T−O bond distances corresponding to the T sites that form the *D4R* were significantly longer than those of the rest of T sites, which was already an indication of the presence of germanium, since the Ge−O distance (1.76 Å) is longer than the Si−O distance (1.61 Å). The electron density clouds on T sites T1-T4 observed in the difference Fourier map (**Fig. S2**) showed unequivocally that Ge was located in the *D4R*. The occupancy factors of the four mixed Si/Ge sites refined to 89 %, 88 %, 27 % and 44 % respectively, which corresponds to Si/Ge= 4.7. This led to a significant improvement of the R values from R_F = 0.122 and R_{wp} = 0.500 (R_{exp} = 0.018), in the pure silicon structure, to R_F = 0.111 and R_{wp} = 0.388.

The mirror plane perpendicular to the *c*-axis divides the *d4R* into two symmetrically equivalent halves, and therefore the percentage of Ge in four of the T sites is necessarily equal to that of their four respective symmetrically equivalent T sites. To check if the Si/Ge fraction is actually different in each T positions of the *D4R*, the mirror plane was removed, reducing the symmetry to *Pba2*. This resulted in an increase in the number of T sites in the asymmetric unit from 16 to 28. After relaxing the structure using a distance-least-squares procedure, refinement of the occupancy parameters of the T sites (again initially modelled as pure Si) showed that Ge is located exclusively at the eight T sites (T1-T8) that form the *D4R* in *Pba2*. It was reasoned that Ge might tend to avoid Ge−O−Ge bonds, and therefore, to get a set of starting values for the Ge occupancy factors in the *D4R*, those of the four Ge atoms that are not connected through a Ge−O−Ge bond were constrained to have the same value. However, when they were subsequently refined freely, they yield values that indicate the existence of these bonds, as will be discussed later. Refinement of the framework coordinates without restraints led to T−O distances for T1-T8 that are consistent with Ge population, *i.e.* the T sites with higher percentage of Ge have longer T−O distances. At this stage the difference Fourier map still showed some electron density clouds on mixed Si/Ge atoms, suggesting that Ge population may have to be higher; however they did not refine to higher values. A closer inspection of the map revealed the presence of some “holes” close to the electron density peaks, which indicated that the position of these Ge atoms was not correct yet. Bearing in mind that the Ge−O distance is significantly longer than the Si−O distance, these discrepancies in the Fourier map could mean that the position of the T atom is slightly different depending on whether there is Si or Ge on that particular case, since a single

(average) T position is being used. A few more cycles of refinement of the structure led to a cleaner Fourier map (the peaks and holes were just 1.5 electron/Å^3 high or deep). Two nonframework-atom positions were found, and they were included as water molecules (modelled as oxygen atoms). Ow(1) is located at the intersection between the 8-ring and 10-ring channel (3.5 Ow(1) per unit cell), within hydrogen-bonding distance from oxygen O19 (Ow(1)−O19 = 2.23 Å). Ow(2) lies in the 12-ring channel (4 Ow(2) per unit cell, 2 per channel), making two close contacts with two framework oxygens (Ow(2)−O1= 2.54 Å and Ow(2)−O37=3.09 Å). This makes a total of 7.5 water molecules per unit cell, probably adsorbed after calcination.

All framework atoms were refined isotropically, and to keep the number of parameters to a minimum, only one displacement parameter was refined for oxygen and one for the T atoms. The thermal parameters of Si/Ge and O refined to values close to 0.01 and 0.02, respectively, so they kept fixed at those values. Geometric restraints were used on the bond distances and angles of the framework atoms through the refinement, but their relative weight with respect to the powder diffraction data was reduced during the course of the refinement. For the mixed Si/Ge sites, the bond distances restraints were adjusted to reflect the amount of Ge at each site. No evidence of Ge with a coordination higher than 4 was observed. The crystallographic data are given in **Table S4**, atomic parameters in **Table S5** and the fit of the profile calculated for this model to the experimental data is shown in **Fig. S3**. Selected interatomic distances and bond angles are given in **Table S6** and **Table S7**.

A similar procedure was followed for the Rietveld refinement of the diffraction data of Ge-poor **IWW**, with lower Ge content. The pattern contains two broad peaks with low intensity that do not belong to **IWW**, which were included in the background (**Fig. S4**) so they do not disturb the refinement. Similarly to what was found for Ge-rich **IWW**, the longer T−O bond distances for T1-T8 and the electron density clouds on these T sites were indicative of the presence of Ge in the *d4R*'s. Their occupancy factors refined to a Si/Ge ratio of *ca.* 6, in good agreement with that found in the chemical analysis. No extraframework species were found. The crystallographic data are given in **Table S8**, atomic parameters in **Table S9** and the fit of the profile calculated for this model to the experimental data is shown in **Fig. S5**. Selected interatomic distances and bond angles are given in **Table S10** and **Table S11**.

In both samples the refined T−O distances are consistent with Ge population, being the T−O average distances for T sites in the *d4R* (T1-T8, see **Fig. S1**) longer than the typical Si−O bond length, and longer for T sites with higher Ge occupancy. The T−O−T bond angles are, on average, ca. 141° if at least one of the T sites is a mixed Si/Ge position, and ca. 151° otherwise. In both samples Ge atoms are located exclusively at the *d4R*'s. This is the preferential location for Ge found in ITQ-22 (Si/Ge = 3.2) when it was first synthesized and solved1, although in that case small amounts of Ge (occupancies less than 11 %) were found in 6 other T sites, and the substitution of Si by Ge in the T sites of the *D4R* is around 55 % (4 T sites because it was solved and refined in *Pbam*). A significantly higher percentage of Ge was found in the *D4R* in Ge-rich IWW (see **Table S5**). Interestingly, one of the 4-rings in the *D4R* (T1-T4 atoms) is formed almost exclusively by Ge (> 90% Ge at each T site), whereas ca. 50 % of the Si has been replaced by Ge in the other 4-ring (T5-T8 atoms in **Fig. S1**). The eight Ge-containing T sites yield a total of 0.94+0.94+0.90+0.98+0.67+0.57+0.69+0.30 = 6.0

Ge sites in the asymmetric unit (i.e. 6.0 × 4 = 24.0 Ge per unit cell), which gives a Si/Ge = 3.7 nicely corresponding to Si/Ge = 3.6 found by the chemical analysis. In Ge-poor IWW, there is a more even distribution of Ge atoms over the 8 T sites of the *d4R* (see **Table S9**), being T1 and T4 the ones with the highest percentage of Ge. These two T sites are in the same 4-ring that is almost entirely Ge−O−Ge in Ge-rich **IWW**. There are 15.8 Ge atoms per unit cell, yielding a Si/Ge = 6.1, in a good agreement with the chemical analysis (Si/Ge = 6.4).

The composition of the *D4R′*s in Ge-rich and Ge-poor **IWW** samples is [6Ge,2Si] and [4Ge,4Si], respectively, which involves the existence of Ge−O−Ge bonds in the *D4R*'s. They have also been observed in IWW in Ref. 1, with Ge occupancy factors bigger than 50 % in six of the eight T sites. The incorporation of Ge is known to favor the crystallization of zeolites that contain *d4R*'s because the increase of the average T−O bond distance and decrease of the T−O−T angle relax the structure and makes it more stable. However, large distortions of the *d4R*'s caused by a high Ge content are expected to decrease the overall stability of the zeolite. Indeed, a linear correlation has been observed between the relative total energy versus the number of Ge−O−Ge linkages present in the corresponding distribution. This means that Ge atoms, from an energetic point of view, tend to locate far from each other[11].

For Ge-rich IWW, a complete 4-ring can be expected to be dissolved (the almost purely Ge one) upon treatment in acid solution, as well as part of the other 4-ring composed by mixed T positions Si/Ge. By contrast, for Ge-poor **IWW** all T sites in the *D4R* are mixed Si/Ge positions and consequently this cage would be partially removed during the acid treatment, *i.e.* the extraction of some of the 8 T atoms rather than disassembly of a complete 4-ring would occur.

Germanium has been found to progressively incorporate into the *D4R* in ITQ-17[12] (**BEC**) to reach four Ge atoms per cage (50 % replacement of Si by Ge in T1, the only T site in the *d4R*), and a further increase of the Ge content favors the substitution of Si atoms at crystallographic sites not in the *d4R*[12]. This is in agreement with ^{19}F MAS NMR spectroscopy results, which provide evidence about the composition of the distribution of Ge among the *D4R*'s, since a low field shift of the ^{19}F signal assigned to F- in the *D4R* has been observed with increasing *n* values [*n*Ge,(8-*n*)Si] in that *D4R*'[12,13]. Besides, computational calculations on ITQ-17 showed that at high Ge content (Si:Ge 1.7 : 1), where the *D4R*'s contain 4 Ge and 4 Si atoms as shown by the Rietveld refinement results, configurations with Ge at the opposite sides of the *d4R* are preferred to avoid the formation of Ge−O−Ge bonds. Configurations with five Ge atoms in one *D4R* where found to be higher in energy, and consequently, less probable, because they involve three Ge−O−Ge linkages[12]. However, a different behavior has been observed for zeolite ITQ-2111. In this case the T1 position in the *D4R* is also preferred, and T2 is only occupied for higher Ge content. However, T3 sites remain purely siliceous even for Si/Ge ratios as low as 1.7. It forces the formation of some Ge−O−Ge bonds, as was confirmed by the presence of a band at −14 ppm in the ^{19}F NMR MAS spectrum, associated with the presence of Ge−O−Ge in Ge-rich *D4R*'s. This was explained by computational simulation results, which show that the incorporation energy involved in the replacement of Si by Ge in T3 is even larger than that required to form Ge−O−Ge pairs. A similar effect in **IWW** structure might explain the presence of Ge−O−Ge bonds in the samples studied in this work and in Ref. 1. Actually, a signal at -2 ppm in the ^{19}F MAS NMR spectrum of ITQ-22 after post-synthesis introduction of F- anions has been assigned to [3Si,5Ge] *D4R*'s[14], which

would confirm the high population of Ge in these cages for this zeolite. This signal has not been observed for zeolites with [4Ge,4Si] (Si/Ge = 1) in the *D4R*. Few examples of *D4R*'s with Si/Ge ratio lower than one can be found in the literature. One of them is the new microporous germanosilicate IM-20, with [5Ge,3Si] *d4R*'s[15], and the germanates analogues of zeolites ABW, ANA, AST, ASV, GIS, SOD, RHO, etc.[16,17,18] From the synthesis and structure analysis of AST-type framework with different Si/Ge ratio, including the Si- and Ge-end members, Wang et al.[19] concluded that Ge is first inserted in the *D4R*'s ordering in a Lowenstein avoidance rule-like pattern, being the 6:2, 4:4 and 2:6 Si:Ge ratios preferred, apart from all Si and all Ge *D4R*'s.

The two samples studied in this work show T−T distances within the *d4R* longer than the 3.1 Å typically found for purely siliceous zeolites like octadecasil (**AST**)[20], ITQ-7 (**ISV**)[21] or ITQ-17 (**BEC**)[12] as expected from the Ge's larger ionic radii. The distances for Ge-poor **IWW** are ca. 3.26 Å except those involving one of the T sites with the highest Ge population (T1 and T4), which are up to 3.34 Å. An increase in Ge content produces a lengthening of the T−T distances in Ge-rich IWW (up to 3.37 Å). They are slightly longer than the 3.2-Å distances of Ge-containing zeolites ITQ-22 (Si/Ge= 4)1 and ITQ-24 (Si/Ge= 7.6, **IWR**)[22] and the pure Ge AST[19].

Refinement of the structures in the space group *Pba2* allowed differences between the two 4-rings (formed by T atoms T1-T4 and T5-T8, see **Fig. S1**) that compose the *D4R* to come to light, since the mirror plane that made them equivalent in *Pbam* has been removed. Apart from an independent occupancy factor for Ge in each of the eight T sites, subtle structural differences were also apparent, because the T−T distances that were equivalent in Pbam are not forced to be equal in *Pba2*. This is more evident for the *D4R*'s face diagonal T1−T4 compared to T2−T3, which differ in ca. 0.20 Å for both samples, and for T6−T3 compared to T5−T4 and T7−T4 compared to T3−T8 in Ge-rich **IWW**, which also differ in 0.20 Å.

Table S1 Examples of conditions used for hydrolysis of Ge-rich IWW (Si/Ge ratio 3.1)

Sample	Acid and its concentration used for hydrolysis			Temperature	Time
	HCl	HNO_3	CH_3COOH		
**1**	0.1M			ambient	24 hours
**2**	0.1M			85˚C	24 hours
**3**	1M			ambient	24 hours
**4**	12M			ambient	48 hours
**5**		0.1M		ambient	24 hours
**6**		1M		ambient	24 hours
**7**			1M	ambient	24 hours

Table S2 Textural properties of **IWW** samples valuated from argon and nitrogen sorption

Sample	Si/Ge (EDX)	Adsorbate					
		Nitrogen *			Argon – low pressure data **		
		BET (m^2/g)	V_{mic} (cm^3/g)	V_{tot} (cm^3/g)	BET (m^2/g)	V_{mic} (cm^3/g)	V_{tot} (cm^3/g)
parent IWW	3.1	439.7	0.163	0.496	416.1	0.169	0.484
hydrolysed calcined (IPC-5)	45.86	143.4	0.041	0.216	123.6	0.030	0.213
surfactant-treated calcined (IPC-5SW calc)	-	300.5	0	0.353	278.5	0.015	0.278
restored IWW	73.38	612.9	0.124	0.493	509.5	0.146	0.450

* BET – calculated from the range p/p_0 0.05-0.20, * V_{tot} – at p/p_0 = 0.98, * V_{mic} – determined from *t*-plot method,** BET – calculated from the range p/p_0 0.05-0.20, ** V_{tot} – at p/p_0 = 0.99, ** V_{mic} – evaluated from DFT method

Table S3 Powder diffraction data collection parameters for Ge-rich and Ge-poor **IWW** samples

Synchrotron facility	SLS
Beamline	Material Science
Diffraction geometry	Debye-Scherrer
Detector	Mythen II Si microstrip
Monochromator	Si 111
Wavelength	1.000 Å
Sample	Rotating 0.3 mm capillary
Nominal step size	0.004 °2θ
Detector positions	4
Time per pattern	24 s
2θ range	1.0−120°2θ (only 2.4−45.0 °2θ was used in the refinement)

Table S4 Crystallographic data from the Rietveld refinement of Ge-rich **IWW**[a]

Chemical composition		$\|H_2O\|_4[Si_{86.2}Ge_{25.8}O_{224}]$
Unit cell		
a		42.0902(9) Å
b		12.9738(4) Å
c		12.6657 (3) Å
Space Group		*Pba2*
Standard peak (*hkl*, 2θ)		3 1 0, 6.02°
Peak range (FWHM)		25
Data points		11037
Contributing reflections		1619
Geometric restraints		336
Si−O	1.61(1)-1.76(1) Å	80
O−Si−O	109.5(2)°	168
Si−O−Si	145(8) °	36
Si/Ge−O[b]	1.70(1)-1.76(1) Å	32
Si/Ge−O−Si/Ge[c]	135(8) °	20
Parameters		
structural		263
profile		8
R_F		0.100
R_{wp}		0.152
R_{exp}		0.036

[a] The numbers given in parentheses are the esd's in the units of the least significant digit given. Each restraint was given a weight equivalent to the reciprocal of its esd.

[b] The T−O bond distance restraint was adjusted to the Ge population at each T site.

[c]The T−O−T bond angle restraint was set to 135 for those angles with at least one T site partially occupied by Ge.

Table S5 Atomic parameters for Ge-rich **IWW**[a,b]

Atom/site	*x*	*y*	*z*	U_{iso}	% Ge[c]
T1	0.3899(2)	0.9157(6)	0.1110(1)	0.01	94(1)
T2	0.8837(2)	0.5679(6)	0.8549(2)	0.01	94(1)
T3	0.4312(2)	0.6955(7)	0.0953(10)	0.01	90(1)
T4	0.9258(2)	0.7814(7)	0.8381(2)	0.01	98(1)
T5	0.3617(2)	0.5645(6)	0.0852(12)	0.01	67(3)
T6	0.8575(2)	0.9144(7)	0.8292(13)	0.01	57(4)
T7	0.3215(2)	0.7806(8)	0.1097(8)	0.01	69(1)
T8	0.8170(2)	0.7037(9)	0.8593(11)	0.01	30(1)
T9	0.3844(2)	0.8965(6)	0.4879(9)	0.01	
T10	0.4201(2)	0.6877(7)	0.4670(10)	0.01	
T11	0.3541(2)	0.5672(7)	0.4673(11)	0.01	
T12	0.3182(2)	0.7753(8)	0.4885(10)	0.01	
T13	0.3529(2)	0.4106(7)	0.2705(10)	0.01	
T14	0.8454(2)	0.0870(7)	0.6670(10)	0.01	
T15	0.3818(2)	0.0592(6)	0.3022(8)	0.01	
T16	0.8802(2)	0.4319(6)	0.6597(8)	0.01	
T17	0.2794(2)	0.8670(8)	0.2924(10)	0.01	
T18	0.7800(2)	0.6427(7)	0.6711(10)	0.01	
T19	0.4647(2)	0.6170(6)	0.2877(10)	0.01	
T20	0.9669(2)	0.8722(5)	0.6527(10)	0.01	
T21	0.2896(2)	0.3035(7)	0.3457(10)	0.01	
T22	0.7875(2)	0.2067(7)	0.5952(10)	0.01	
T23	0.3086(2)	0.0764(8)	0.3387(10)	0.01	
T24	0.8077(2)	0.4292(7)	0.5903(10)	0.01	
T25	0.4074(2)	0.2756(7)	0.3581(8)	0.01	
T26	0.9045(2)	0.2135(7)	0.6066(9)	0.01	
T27	0.4680(2)	0.3923(6)	0.3597(9)	0.01	
T28	0.9650(2)	0.0992(6)	0.6087(9)	0.01	
O1	0.3963(2)	1.0223(9)	0.1918(8)	0.02	
O2	0.4160(3)	0.8135(9)	0.1447(11)	0.02	
O3	0.3969(4)	0.9564(10)	-0.0177(5)	0.02	
O4	0.3499(4)	0.8754(11)	0.1252(12)	0.02	
O5	0.8866(3)	0.4549(8)	0.7819(8)	0.02	
O6	0.8435(4)	0.6076(11)	0.8566(16)	0.02	
O7	0.9081(3)	0.6637(10)	0.7998(10)	0.02	
O8	0.4642(4)	0.6582(9)	0.1688(11)	0.02	
O9	0.4427(2)	0.7139(14)	-0.0353(9)	0.02	
O10	0.4015(3)	0.5980(11)	0.1008(18)	0.02	
O11	0.9564(3)	0.8065(9)	0.7514(12)	0.02	
O12	0.8969(3)	0.8800(11)	0.8265(17)	0.02	
O13	0.3536(2)	0.4543(8)	0.1525(12)	0.02	

O14	0.3379(3)	0.6615(9)	0.1330(16)	0.02	
O15	0.3532(4)	0.5434(12)	-0.0450(12)	0.02	
O16	0.8413(5)	0.0207(10)	0.7735(15)	0.02	
O17	0.8333(4)	0.8100(9)	0.8072(6)	0.02	
O18	0.2908(4)	0.7992(10)	0.1945(2)	0.02	
O19	0.3069(3)	0.7843(10)	-0.0154(9)	0.02	
O20	0.7848(3)	0.6709(11)	0.7927(10)	0.02	
O21	0.4114(2)	0.8083(8)	0.4779(14)	0.02	
O22	0.3849(3)	0.9660(10)	0.3840(11)	0.02	
O23	0.3910(4)	0.9692(10)	0.5896(11)	0.02	
O24	0.3498(4)	0.8448(11)	0.4993(15)	0.02	
O25	0.4470(2)	0.6598(11)	0.5507(12)	0.02	
O26	0.3888(3)	0.6178(11)	0.4903(14)	0.02	
O27	0.4342(2)	0.6630(13)	0.3507(11)	0.02	
O28	0.3279(3)	0.6582(9)	0.4593(12)	0.02	
O29	0.3443(3)	0.4886(12)	0.5634(14)	0.02	
O30	0.3552(3)	0.5049(10)	0.3553(13)	0.02	
O31	0.2946(4)	0.8221(13)	0.4000(12)	0.02	
O32	0.2999(3)	0.7776(10)	0.5995(11)	0.02	
O33	0.3826(3)	0.3342(11)	0.2838(11)	0.02	
O34	0.3205(3)	0.3480(11)	0.2870(12)	0.02	
O35	0.8780(3)	0.1503(12)	0.6706(11)	0.02	
O36	0.8172(3)	0.1684(10)	0.6614(12)	0.02	
O37	0.4027(3)	0.1541(8)	0.3444(11)	0.02	
O38	0.3444(3)	0.0918(13)	0.2905(14)	0.02	
O39	0.9013(2)	0.3343(8)	0.6281(12)	0.02	
O40	0.8427(3)	0.4066(12)	0.6406(13)	0.02	
O41	0.2907(2)	0.9846(8)	0.2763(13)	0.02	
O42	0.2413(3)	0.8605(10)	0.2988(12)	0.02	
O43	0.7919(2)	0.5263(8)	0.6506(13)	0.02	
O44	0.7433(3)	0.6514(9)	0.6425(13)	0.02	
O45	0.4636(2)	0.4929(6)	0.2867(10)	0.02	
O46	0.4968(4)	0.6534(11)	0.3444(13)	0.02	
O47	0.9630(2)	0.9934(6)	0.6752(11)	0.02	
O48	1.0026(4)	0.8462(9)	0.6311(12)	0.02	
O49	0.2881(2)	0.1812(8)	0.3244(15)	0.02	
O50	0.2918(3)	0.3249(12)	0.4719(10)	0.02	
O51	0.7850(3)	0.3299(8)	0.6069(15)	0.02	
O52	0.3108(3)	0.0467(12)	0.4641(10)	0.02	
O53	0.4427(4)	0.3069(10)	0.3247(12)	0.02	
O54	0.4013(2)	0.3079(10)	0.4806(8)	0.02	
O55	0.9380(3)	0.1755(8)	0.6462(11)	0.02	
O56	0.4622(2)	0.4231(10)	0.4828(9)	0.02	
Ow(1)	0.7136(2)	0.0198(9)	0.0000	0.04	88(4)[d]

Ow(2)	0.4538(3)	0.0903(10)	0.1791(9)	0.04	100[d]

[a]: T is used to designate the mixed Si/Ge T sites. The multiplicity of all atoms is 1.

[b]: Numbers in parentheses are the standard deviations (esd's) in the units of the least significant digit given. Each restraint was given a weight equivalent to the reciprocal of its esd. Values without an esd were not refined.

[c]: The % of Si at each T site is the difference to 100 %. The rest of the T sites have been found to be purely siliceous.

[d]: Occupancy factors of the water molecules.

Table S6 Selected interatomic distances (Å) for Ge-rich **IWW**[a]

T1	− O1	1.74	Si11	− O26	1.63	Si21	− O34	1.60
T1	− O2	1.77	Si11	− O28	1.62	Si21	− O42	1.61
T1	− O3	1.74	Si11	− O29	1.64	Si21	− O49	1.61
T1	− O4	1.77	Si11	− O30	1.63	Si21	− O50	1.62
T2	− O3	1.73	Si12	− O24	1.61	Si22	− O36	1.59
T2	− O5	1.74	Si12	− O28	1.62	Si22	− O44	1.60
T2	− O6	1.77	Si12	− O31	1.62	Si22	− O50	1.63
T2	− O7	1.76	Si12	− O32	1.60	Si22	− O51	1.61
T3	− O2	1.77	Si13	− O13	1.60	Si23	− O38	1.64
T3	− O8	1.74	Si13	− O30	1.63	Si23	− O41	1.62
T3	− O9	1.74	Si13	− O33	1.60	Si23	− O49	1.62
T3	− O10	1.78	Si13	− O34	1.60	Si23	− O52	1.64
T4	− O7	1.77	Si14	− O16	1.61	Si24	− O40	1.63
T4	− O9	1.76	Si14	− O29	1.64	Si24	− O43	1.62
T4	− O11	1.72	Si14	− O35	1.60	Si24	− O51	1.62
T4	− O12	1.77	Si14	− O36	1.59	Si24	− O52	1.64
T5	− O10	1.74	Si15	− O1	1.60	Si25	− O33	1.60
T5	− O13	1.70	Si15	− O22	1.60	Si25	− O37	1.60
T5	− O14	1.72	Si15	− O37	1.60	Si25	− O53	1.60
T5	− O15	1.71	Si15	− O38	1.64	Si25	− O54	1.63
T6	− O12	1.72	Si16	− O5	1.60	Si26	− O35	1.60
T6	− O15	1.69	Si16	− O23	1.63	Si26	− O39	1.60
T6	− O16	1.69	Si16	− O39	1.60	Si26	− O54	1.62
T6	− O17	1.72	Si16	− O40	1.63	Si26	− O55	1.58
T7	− O4	1.73	Si17	− O18	1.59	Si27	− O45	1.61
T7	− O14	1.72	Si17	− O31	1.62	Si27	− O46	1.61
T7	− O18	1.70	Si17	− O41	1.61	Si27	− O53	1.60
T7	− O19	1.70	Si17	− O42	1.61	Si27	− O56	1.63
T8	− O6	1.67	Si18	− O20	1.60	Si28	− O47	1.61
T8	− O17	1.68	Si18	− O32	1.61	Si28	− O48	1.56
T8	− O19	1.65	Si18	− O43	1.61	Si28	− O55	1.58
T8	− O20	1.65	Si18	− O44	1.59	Si28	− O56	1.63
Si9	− O21	1.62	Si19	− O8	1.60			

Si9	− O22	1.60	Si19	− O27	1.63
Si9	− O23	1.62	Si19	− O45	1.61
Si9	− O24	1.61	Si19	− O46	1.60
Si10	− O21	1.61	Si20	− O11	1.58
Si10	− O25	1.59	Si20	− O25	1.59
Si10	− O26	1.63	Si20	− O47	1.61
Si10	− O27	1.62	Si20	− O48	1.56

[a]: T is used to designate the mixed Si/Ge T sites.

Table S7 Selected bond angles (º) for Ge-rich **IWW**

O−T−O	min	105.8			
	max	111.4			
	avg	109.4			
T−O−T[a]	min	127.4	T−O−T[b]	min	136.2
	max	149.0		max	166.3
	avg	141.0		avg	149.5

[a] mixed Si/Ge T sites (T1-T8).
[b] purely siliceous T sites (T9-T28).

Table S8 Crystallographic data from the Rietveld refinement of Ge-poor **IWW**[a]

Chemical composition		[$Si_{96.2}Ge_{15.8}O_{224}$]
Unit cell		
a		41.9986(7) Å
b		12.9554(3) Å
c		12.6100 (2) Å
Space Group		*Pba2*
Standard peak (*hkl*, 2θ)		2 0 0, 2.73°
Peak range (FWHM)		15
Data points		10984
Contributing reflections		1716
Geometric restraints		336
Si−O	1.61(1)-1.76(1) Å	80
O−Si−O	109.5(2)°	168
Si−O−Si	145(8) °	36
Si/Ge−O[b]	1.70(1)-1.76(1) Å	32
Si/Ge−O−Si/Ge[c]	135(8) °	20
Parameters		
structural		262
profile		8
R_F		0.078
R_{wp}		0.076
R_{exp}		0.029

[a] The numbers given in parentheses are the esd's in the units of the least significant digit given. Each restraint was given a weight equivalent to the reciprocal of its esd.
[b] The T−O bond distance restraint was adjusted to the Ge population at each T site.

[c]The T−O−T bond angle restraint was set to 135 º for those angles with at least one T site partially occupied by Ge.

Table S9 Atomic parameters for Ge-poor **IWW** (55)[a,b]

Atom/site	*x*	*Y*	*z*	U_{iso}	% Ge[a]
T1	0.3869(2)	0.9183(5)	0.1941(5)	0.01	80(2)
T2	0.8833(2)	0.5730(5)	0.9343(5)	0.01	35(2)
T3	0.4259(1)	0.6980(6)	0.1881(10)	0.01	55(2)
T4	0.9227(2)	0.7860(6)	0.9239(10)	0.01	71(2)
T5	0.3580(2)	0.5749(5)	0.1823(10)	0.01	53(2)
T6	0.8566(2)	0.9128(6)	0.9245(11)	0.01	22(2)
T7	0.3192(2)	0.7897(6)	0.1977(7)	0.01	45(3)
T8	0.8174(2)	0.7048(8)	0.9392(7)	0.01	35(2)
T9	0.3841(2)	0.8965(5)	0.5599(7)	0.01	
T10	0.4195(1)	0.6904(6)	0.5590(10)	0.01	
T11	0.3544(2)	0.5689(5)	0.5610(11)	0.01	
T12	0.3193(2)	0.7762(6)	0.5641(8)	0.01	
T13	0.3539(2)	0.4163(6)	0.3601(9)	0.01	
T14	0.8451(2)	0.0882(6)	0.7576(10)	0.01	
T15	0.3820(2)	0.0606(5)	0.3813(6)	0.01	
T16	0.8792(2)	0.4380(5)	0.7434(6)	0.01	
T17	0.2763(1)	0.8662(6)	0.3834(8)	0.01	
T18	0.7828(2)	0.6354(7)	0.7507(8)	0.01	
T19	0.4625(2)	0.6160(5)	0.3760(10)	0.01	
T20	0.9661(2)	0.8715(5)	0.7424(10)	0.01	
T21	0.2927(2)	0.3033(5)	0.4396(9)	0.01	
T22	0.7866(2)	0.1999(6)	0.6810(9)	0.01	
T23	0.3090(1)	0.0724(6)	0.4376(10)	0.01	
T24	0.8085(1)	0.4237(6)	0.6834(10)	0.01	
T25	0.4071(2)	0.2799(5)	0.4378(8)	0.01	
T26	0.9028(2)	0.2222(6)	0.6857(8)	0.01	
T27	0.4690(2)	0.3916(5)	0.4460(8)	0.01	
T28	0.9645(1)	0.0998(5)	0.6929(9)	0.01	
O1	0.3946(2)	1.0264(8)	0.2678(6)	0.02	
O2	0.4151(4)	0.8217(9)	0.2213(15)	0.02	
O3	0.3883(5)	0.9538(8)	0.0620(4)	0.02	
O4	0.3491(3)	0.8731(12)	0.2265(14)	0.02	
O5	0.8882(3)	0.4661(7)	0.8634(6)	0.02	
O6	0.8465(3)	0.6193(12)	0.9142(14)	0.02	
O7	0.9103(4)	0.6610(9)	0.8973(13)	0.02	
O8	0.4590(2)	0.6617(8)	0.2575(11)	0.02	
O9	0.4337(3)	0.6936(16)	0.0554(10)	0.02	
O10	0.3955(3)	0.6148(13)	0.2153(16)	0.02	
O11	0.9551(2)	0.8124(8)	0.8467(12)	0.02	
O12	0.8922(3)	0.8699(14)	0.8904(16)	0.02	
O13	0.3495(2)	0.4647(7)	0.2452(12)	0.02	

O14	0.3314(4)	0.6670(8)	0.2191(17)	0.02
O15	0.3555(5)	0.5538(11)	0.0502(10)	0.02
O16	0.8473(3)	0.0208(9)	0.8640(14)	0.02
O17	0.8293(4)	0.8230(9)	0.9011(18)	0.02
O18	0.2875(2)	0.8137(10)	0.2747(10)	0.02
O19	0.3091(3)	0.8032(20)	0.0687(7)	0.02
O20	0.7851(2)	0.6722(9)	0.8712(8)	0.02
O21	0.4112(3)	0.8105(6)	0.5612(12)	0.02
O22	0.3908(3)	0.9731(9)	0.4648(9)	0.02
O23	0.3841(4)	0.9611(8)	0.6692(8)	0.02
O24	0.3503(2)	0.8433(1)	0.5406(13)	0.02
O25	0.4464(2)	0.6696(9)	0.6437(13)	0.02
O26	0.3882(2)	0.6231(10)	0.5862(14)	0.02
O27	0.4331(2)	0.6577(12)	0.4456(12)	0.02
O28	0.3274(3)	0.6561(7)	0.5534(12)	0.02
O29	0.3453(3)	0.4883(10)	0.6550(14)	0.02
O30	0.3559(3)	0.5076(10)	0.4487(13)	0.02
O31	0.2916(2)	0.8059(10)	0.4824(10)	0.02
O32	0.3074(3)	0.7993(12)	0.6812(9)	0.02
O33	0.3859(2)	0.3499(9)	0.3616(9)	0.02
O34	0.3242(2)	0.3421(8)	0.3812(11)	0.02
O35	0.8747(3)	0.1675(9)	0.7522(10)	0.02
O36	0.8126(2)	0.1530(10)	0.7604(11)	0.02
O37	0.4003(3)	0.1624(10)	0.4129(11)	0.02
O38	0.3437(2)	0.0798(6)	0.3799(12)	0.02
O39	0.9018(2)	0.3454(11)	0.7041(10)	0.02
O40	0.8426(2)	0.4012(7)	0.7380(12)	0.02
O41	0.2879(2)	0.9838(10)	0.3838(12)	0.02
O42	0.2381(2)	0.8603(6)	0.3905(11)	0.02
O43	0.7914(3)	0.5159(8)	0.7442(13)	0.02
O44	0.7475(2)	0.6528(7)	0.7077(11)	0.02
O45	0.4626(4)	0.4914(8)	0.3724(10)	0.02
O46	0.4956(2)	0.6529(5)	0.4279(12)	0.02
O47	0.9608(4)	0.9934(10)	0.7567(10)	0.02
O48	1.0027(2)	0.8475(5)	0.7231(11)	0.02
O49	0.2901(2)	0.1802(11)	0.4260(13)	0.02
O50	0.2956(3)	0.3313(6)	0.5625(9)	0.02
O51	0.7862(2)	0.3232(10)	0.6915(14)	0.02
O52	0.3137(3)	0.0451(6)	0.5611(10)	0.02
O53	0.4438(2)	0.3040(10)	0.4164(12)	0.02
O54	0.3985(3)	0.3029(7)	0.5607(8)	0.02
O55	0.9363(2)	0.1773(8)	0.7248(11)	0.02
O56	0.4641(3)	0.4235(9)	0.5679(9)	0.02

[a]: T is used to designate the mixed Si/Ge T sites. The multiplicity of all atoms is 1

[b]: Numbers in parentheses are the standard deviations (esd's) in the units of the least significant digit given. Each restraint was given a weight equivalent to the reciprocal of its esd. Values without an esd were not refined.
[c]: The % of Si at each T site is the difference to 100 %. The rest of the T sites have been found to be purely siliceous.

Table S10 Selected interatomic distances (Å) for Ge-poor **IWW** [a]

T1	− O1	1.71	Si11	− O26	1.62	Si21	− O34	1.59
T1	− O2	1.75	Si11	− O28	1.60	Si21	− O42	1.61
T1	− O3	1.73	Si11	− O29	1.63	Si21	− O49	1.61
T1	− O4	1.74	Si11	− O30	1.62	Si21	− O50	1.60
T2	− O3	1.66	Si12	− O24	1.59	Si22	− O36	1.60
T2	− O5	1.66	Si12	− O28	1.60	Si22	− O44	1.59
T2	− O6	1.68	Si12	− O31	1.60	Si22	− O50	1.59
T2	− O7	1.67	Si12	− O32	1.59	Si22	− O51	1.60
T3	− O2	1.72	Si13	− O13	1.59	Si23	− O38	1.63
T3	− O8	1.71	Si13	− O30	1.63	Si23	− O41	1.60
T3	− O9	1.71	Si13	− O33	1.60	Si23	− O49	1.61
T3	− O10	1.71	Si13	− O34	1.60	Si23	− O52	1.61
T4	− O7	1.73	Si14	− O16	1.60	Si24	− O40	1.61
T4	− O9	1.74	Si14	− O29	1.63	Si24	− O43	1.59
T4	− O11	1.71	Si14	− O35	1.61	Si24	− O51	1.61
T4	− O12	1.73	Si14	− O36	1.60	Si24	− O52	1.61
T5	− O10	1.71	Si15	− O1	1.59	Si25	− O33	1.59
T5	− O13	1.67	Si15	− O22	1.59	Si25	− O37	1.58
T5	− O14	1.70	Si15	− O37	1.58	Si25	− O53	1.60
T5	− O15	1.69	Si15	− O38	1.63	Si25	− O54	1.62
T6	− O12	1.65	Si16	− O5	1.60	Si26	− O35	1.61
T6	− O15	1.64	Si16	− O23	1.62	Si26	− O39	1.61
T6	− O16	1.64	Si16	− O39	1.61	Si26	− O54	1.62
T6	− O17	1.66	Si16	− O40	1.61	Si26	− O55	1.60
T7	− O4	1.69	Si17	− O18	1.60	Si27	− O45	1.61
T7	− O14	1.69	Si17	− O31	1.61	Si27	− O46	1.61
T7	− O18	1.68	Si17	− O41	1.60	Si27	− O53	1.59
T7	− O19	1.69	Si17	− O42	1.61	Si27	− O56	1.61
T8	− O6	1.68	Si18	− O20	1.59	Si28	− O47	1.60
T8	− O17	1.68	Si18	− O32	1.60	Si28	− O48	1.58
T8	− O19	1.67	Si18	− O43	1.59	Si28	− O55	1.60
T8	− O20	1.66	Si18	− O44	1.59	Si28	− O56	1.60
Si9	− O21	1.59	Si19	− O8	1.61			
Si9	− O22	1.58	Si19	− O27	1.61			
Si9	− O23	1.61	Si19	− O45	1.61			
Si9	− O24	1.60	Si19	− O46	1.61			
Si10	− O21	1.60	Si20	− O11	1.59			

Si10	− O25	1.58	Si20	− O25	1.59
Si10	− O26	1.61	Si20	− O47	1.61
Si10	− O27	1.60	Si20	− O48	1.59

[a]: T is used to designate the mixed Si/Ge T sites.

Table S11 Selected bond angles (°) for Ge-poor **IWW**

O−T−O	min	107.3			
	max	111.4			
	avg	109.4			
T−O−T[a]	min	126.2	T−O−T[b]	min	137.6
	max	152.2		max	166.5
	avg	141.9		avg	153.7

[a] mixed Si/Ge T sites (T1-T8).
[b] purely siliceous T sites (T9-T28).

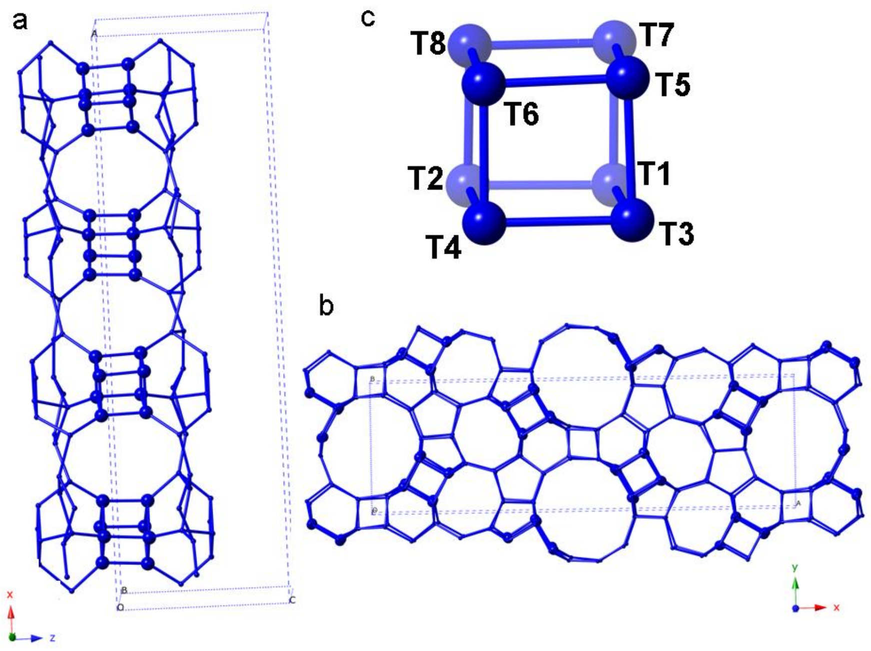

Fig. S1 The framework structure of ITQ-22 (**IWW** framework type). (a) The [010] projection showing the double-four-rings (T atoms represented with balls); (b) the [001] projection showing the 12- and 8-ring channels and (c) T atoms in the double-four-rings. Oxygen atoms have been omitted for clarity.

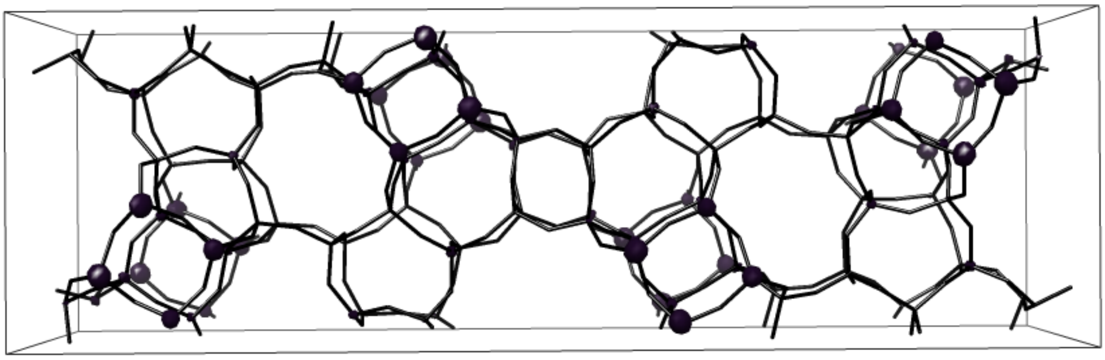

Fig. S2 Fourier differences map for Ge-rich **IWW** with all T atoms as Si. Electron density clouds appear on top of the eight T sites in the *d4R*, indicating the presence of Ge.

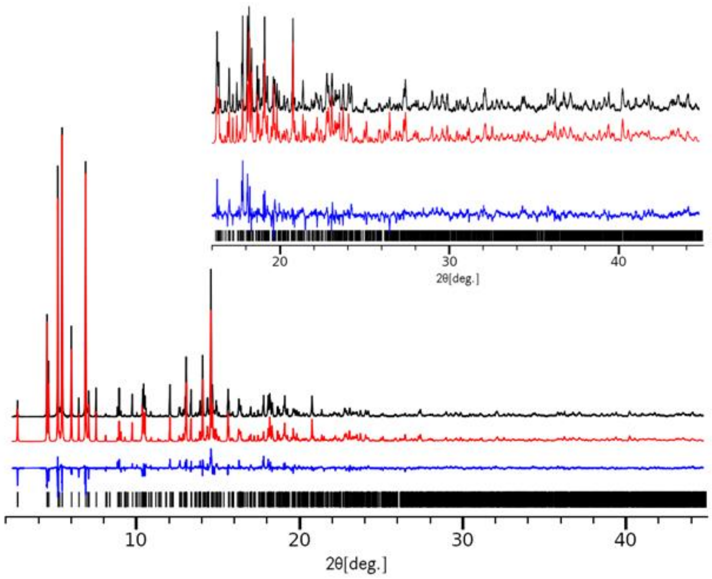

Fig. S3 The observed (black), calculated (red) and difference (blue) profiles for the Rietveld refinement of Ge-rich **IWW**. The data in the inset have been scaled up by a factor of 6 to show more detail at high angles. The tick mark indicate the positions of the reflections.

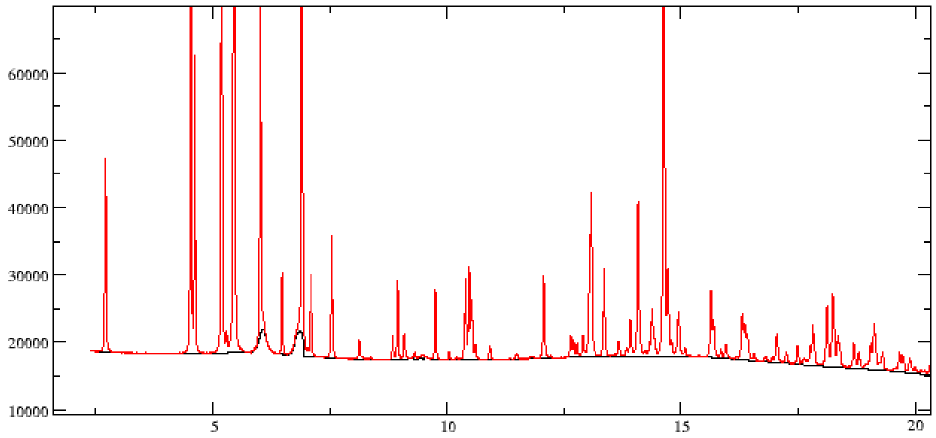

Fig. S4 Background correction for the powder diffraction pattern of the Ge-poor **IWW**. It is an interpolation of points selected by eye and adjusted manually during the course of the refinement. The humps under the two peaks at ca. 6 º correspond to an unidentified impurity that was included in the background so it does not disturb the refinement.

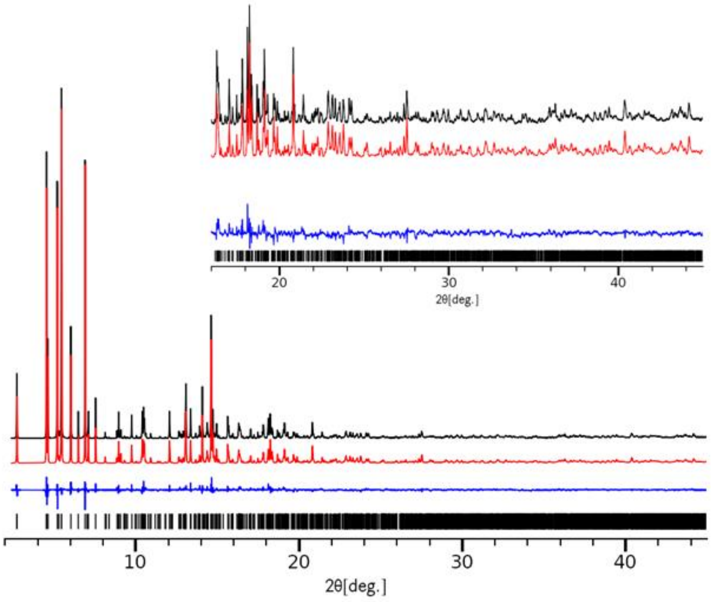

Fig. S5 The observed (black), calculated (red) and difference (blue) profiles for the Rietveld refinement of the Ge-poor **IWW**. The data in the inset have been scaled up by a factor of 6 to show more detail at high angles. The tick marks indicate the positions of the reflections.

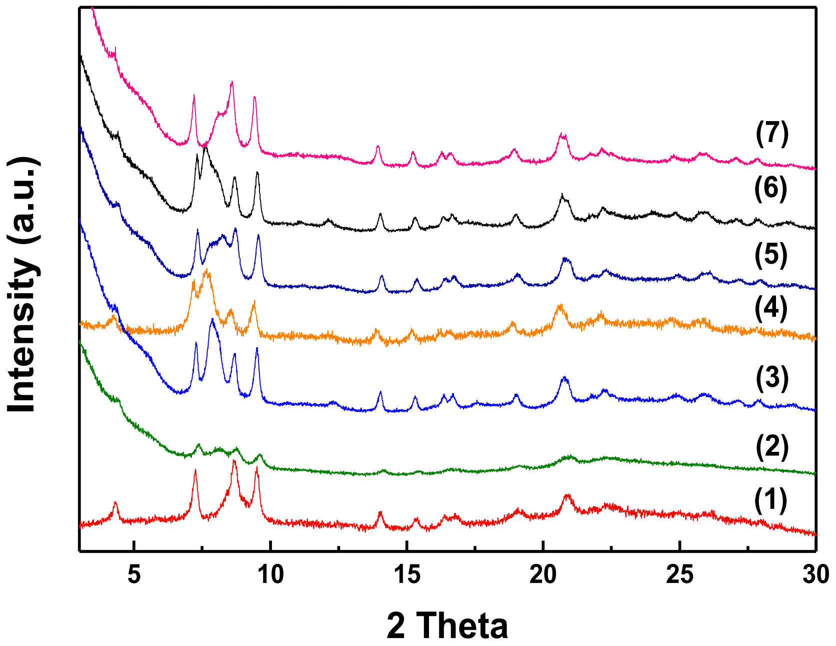

Fig. S6 XRD patterns of IPC-5P samples prepared by hydrolysis of Ge-rich **IWW** (Si/Ge 3.1) under various conditions described in **Table S1.**

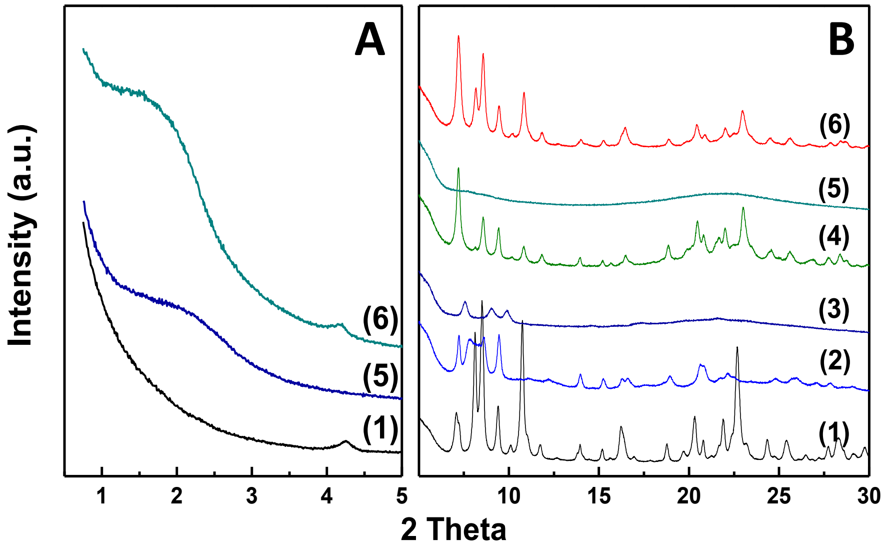

Fig. S7 Small-angle (**A**) and wide-angle (**B**) XRD patterns of parent Ge-rich **IWW** (Si/Ge 3.1) **(1)**, hydrolysed IPC-5P **(2)**, its surfactant-treated IPC-5SW **(4)**, and the surfactant-treated material intercalated with diethoxydimethylsilane and calcined, restored IWW **(6)**. Part of the hydrolysed sample was calcined (IPC-5) **(3)**, as well as part of the surfactant-treated sample (IPC-1SW calc) **(5)**. Note that the high background at low 2 theta angles in the wide angle patterns (B) is caused by instrumental effects (large slits). The low angle region of the small angle patterns (A) show the development of the peaks from the mesopores after treatment.

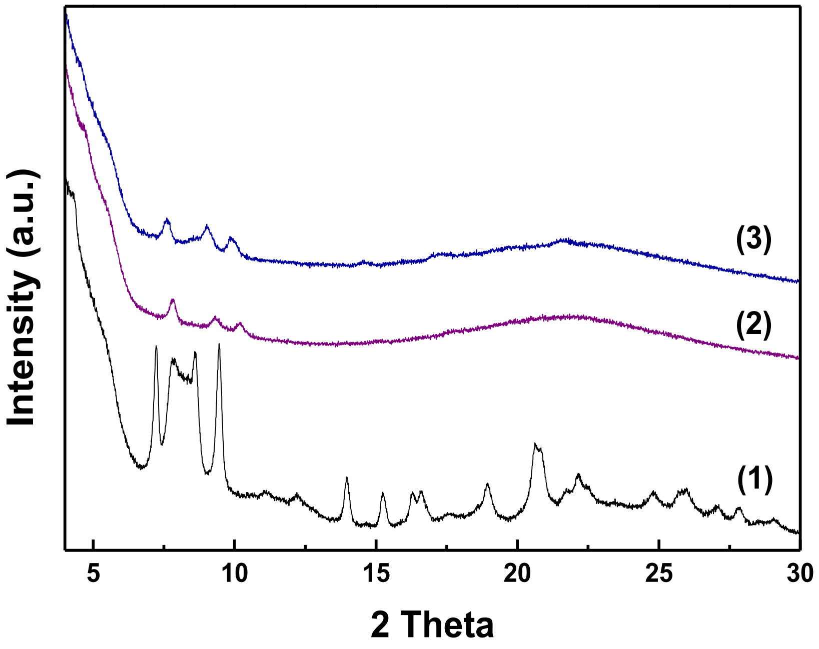

Fig. S8 XRD patterns of hydrolysed Ge-rich **IWW (1)**, after intercalation treatment with octylamine (calcined) **(2)**, and after direct intercalation of diethoxydimethylsilane (calcined) **(3)**.

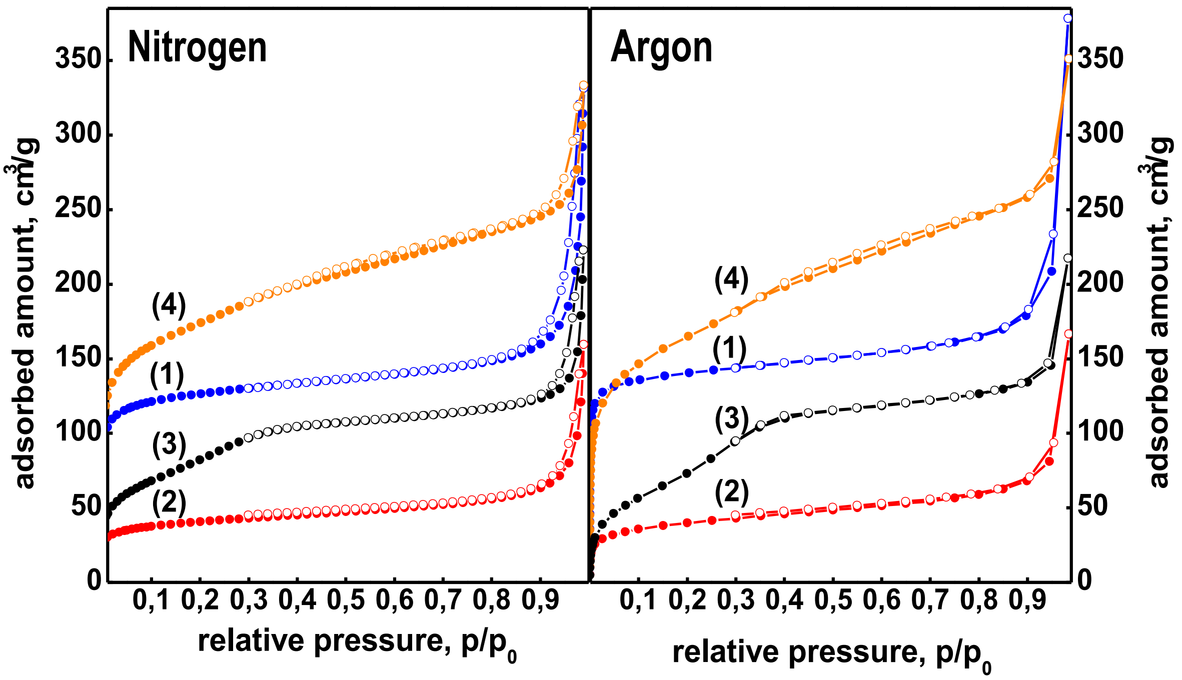

Fig. S9 Adsorption isotherms of nitrogen measured at 77K (left) and argon measured at 87K (right). Parent calcined **IWW** zeolit (Si/Ge 3.1) **(1)**, hydrolysed and calcined IPC-5 **(2)**, surfactant-treated and calcined IPC-5SW calc **(3)**, and surfactant-treated-intercalated and calcined, restored **IWW (4)**. The open points denote desorption. Although the N_2 and Ar isotherms show the same trend for each sample, the values of their BET surface area, micropore volume and others can slightly differ, which is caused by different parameters of both adsorptives (see results in **Table S2**).

[1] A. Corma, F. Rey, S. Valencia, J. L. Jorda, J. Rius, *Nat Mater* **2003**, *2*, 493-497.

[2] A. Bergamaschi, A. Cervellino, R. Dinapoli, F. Gozzo, B. Henrich, I. Johnson, P. Kraft, A. Mozzanica, B. Schmitt, X. Shi, *Nucl. Instrum. Methods Phys. Res., Sect. A* **2009**, *604*, 136-139.

[3] C. Baerlocher, *Acta Crystallogr.* **1984**, *40*, C368-370.

[4] CrystalMaker Software Ltd., O., UK, http://www.crystalmaker.com.

[5] Graesslin, J., Ph.D. Thesis, ETH Zuerich, **2013**.

[6] S. Brunauer, P. H. Emmet, E. Teller, *J. Am. Chem. Soc.* **1938**, *62*, 309-319.

[7] B. C. Lippens, J. H. de Boer, *J. Catal.* **1965**, *4*, 319-323.

[8] E. P. Barret, L. G. Joyner, P. B. Halenda, *J. Am. Chem. Soc.* **1951**, *73*, 373-380.

[9] P. E.Werner, L. Eriksson, M. Westdahl, *J. Appl. Crystallogr.* **1985**, *18*, 367-370.

[10] B. H. Toby, *J. Appl. Crystallogr.* **2005**, *38*, 1040-1041.

[11] T. Blasco, A. Corma, M. J. Diaz-Cabanas, F. Rey, J. Rius, G. Sastre, J. A. Vidal-Moya, *J. Am. Chem. Soc.* **2004**, *126*, 13414-13423.

[12] G. Sastre, J. A. Vidal-Moya, T. Blasco, J. Rius, J. L. Jorda, M. T. Navarro, F. Rey, A. Corma, *Angew. Chem., Int. Ed.***2002**, *41*, 4722-4726.

[13] J. A. Vidal-Moya, T. Blasco, F. Rey, A. Corma, M. Puche, *Chem. Mater.* **2003**, *15*, 3961-3963.

[14] X. L. Liu, U. Ravon, F. Bosselet, G. Bergeret, A. Tuel, *Chem. Mater.***2002**, *24*, 3016-3021.

[15] M. Dodin, J.-L. Paillaud, Y. Lorgouilloux, P. Caullett, E. Elkaim, N. Bats, *J. Am. Chem. Soc.* **2010**, *132*, 10221-20223.

[16] C. M. Baerlocher, D. H. Olson, *Atlas of Zeolite Framework Types*; Elsevier: Amsterdam, **2007**.

[17] X. H. Bu, P. Y. Feng, G. D. Stucky, *Chem. Mater.* **1999**, *11*, 3423-3424.

[18] M. E. Fleet, *Acta Crystallogr. C* **1989**, *45*, 843-847.

[19] Y. X. Wang, J. Q. Song, H. Gies, *Solid State Sci.* **2003**, *5*, 1421-1433.

[20] P. Caullet, J. L. Guth, J. Hazm, J. M. Lamblin, H. Gies, *Eur. J. Solid State Inorg. Chem.* **1991**, *28*, 345-348.

[21] L. A. Villaescusa, P. A. Barrett, M. A. Camblor, *Angew. Chem. Int. Ed.* **1999**, *38*, 1997-2000.

[22] R. Castaneda, A.Corma, V. Fornes, F. Rey, J. Rius, *J. Am. Chem. Soc.* **2003**, *125*, 7820-7821.
